# Supplementary material for: Content-rich biological network constructed by mining PubMed abstracts
Source: BMC Bioinformatics. 2004 Oct 8;5:147. doi: 10.1186/1471-2105-5-147 (PMC528731; doi:10.1186/1471-2105-5-147)
Supplement: Additional File 2 — The original results of the above study (non-essential files are deleted to keep the file size under the limit set by BMC bioinformatics). [file 1471-2105-5-147-S2.bz2 › chilibotAdditionalFile2/dip20/698ID9287012E18025/html/FTSZ_FTSA.html]

 


 **FTSZ** and **FTSA** 
  
Found 107 abstracts in PubMed, retrieved 20.  
 

 What does Google say? 
 PDF only 
| .edu only 

---

**Interactive relationship** (e.g. stimulation, inhibition, etc)

**Neutral relationship**- Specific regions were identified which abrogated  **FtsZ**   **FtsZ**  binding but unaffected the  **FtsA**   **FtsZ**  interaction.  Ref: 11394911 Biochem Biophys Res Commun, 2001
- As in other organisms  **FtsA**  is probably recruited immediately after  **FtsZ** .  Ref: 11298280 Mol Microbiol, 2001
- These results suggest that the molecular mechanism of the  **FtsA** \* bypass is to stabilize  **FtsZ**  assembly via a parallel pathway.  Ref: 12634424 Proc Natl Acad Sci U S A, 2003
- These mutant  **FtsZ**  proteins were analyzed for their interaction with both wildtype  **FtsZ**  and  **FtsA**  using the yeast two hybrid assay.  Ref: 11394911 Biochem Biophys Res Commun, 2001
- The septal ring in Escherichia coli consists of at least nine essential gene products whose order of assembly resembles a mostly linear dependency pathway  **FtsA**  and ZipA directly bind  **FtsZ**  polymers at the prospective division site, followed by the sequential addition of FtsK, FtsQ, FtsL, FtsW, FtsI, and FtsN.  Ref: 11948172 J Bacteriol, 2002
- This suggests that ATP hydrolysis by  **FtsA**  is required, together with GTP hydrolysis by  **FtsZ** , for cell division in B. subtilis and possibly in most eubacteria.  Ref: 11298280 Mol Microbiol, 2001
- **FtsA**  and ZipA localized into a division ring following the same pattern and time course as  **FtsZ** .  Ref: 12754232 J Bacteriol, 2003
- ZipA and  **FtsA**  are essential division proteins in Escherichia coli that are recruited to the division site by interaction with  **FtsZ** .  Ref: 11847116 EMBO J, 2002
- **FtsA**  is present in much higher quantities in B. subtilis than in Escherichia coli, with approximately one molecule of  **FtsA**  for five of  **FtsZ** .  Ref: 11298280 Mol Microbiol, 2001

**Non-interactive relationship** (e.g. studied together, co-existance, homology, etc.)

- The organization of the two chromosomal regions in P. aeruginosa and E. coli is remarkably similar with the following gene order pbp3 pbpB, murE, murF, mraY, murD, ftsW, murG, murC, ddlB, ftsQ,  **ftsA** ,  **ftsZ** , and envA LpxC.  Ref: 11281713 Protein Expr Purif, 2001
- Concentration and Assembly of the Division Ring Proteins  **FtsZ** ,  **FtsA** , and ZipA during the Escherichia coli Cell Cycle.  Ref: 12754232 J Bacteriol, 2003
- Examination of cells expressing different combinations of the fusions indicated that  **FtsA** , FtsQ, FtsL and FtsI co localize with  **FtsZ**  in filaments depleted of FtsN.  Ref: 11703663 Mol Microbiol, 2001
- We also constructed translational fusions of  **FtsZ** ,  **FtsA** , FtsQ, FtsL and FtsI to enhanced cyan or yellow fluorescent protein ECFP or EYFP respectively, GFP variants with different fluorescence spectra.  Ref: 11703663 Mol Microbiol, 2001
- Expression of ftsA1p, the  **ftsA**   **ftsZ**  promoter submitted to RcsB stimulation, is greatly increased in mucM mutants, and this expression is dependent on RcsB and ftsA1p.  Ref: 11425476 FEMS Microbiol Lett, 2001
- By the coexpression of the E. coli  **ftsA**  and  **ftsZ**  genes, which encode key proteins in cell division, growth of recombinant strains as well as production of human leptin and human insulin like growth factor I was improved.  Ref: 12571061 Appl Environ Microbiol, 2003
- Zonal synthesis can be triggered in  **ftsA** , ftsQ and ftsI pbpB division mutants growing as filaments at the restrictive temperature, but not in  **ftsZ**  mutant strains.  Ref: 11782501 Microbiology, 2002
- Mucoidy results from the activation of cps genes and mecillinam resistance from  **ftsA**   **ftsZ**  overexpression, both traits caused by the increased activity of the RcsB effector.  Ref: 11425476 FEMS Microbiol Lett, 2001
- The concentration of the cell division proteins  **FtsZ** ,  **FtsA** , and ZipA and their assembly into a division ring during the Escherichia coli B r K cell cycle have been measured in synchronous cultures obtained by the membrane elution technique.  Ref: 12754232 J Bacteriol, 2003
- Among partners of  **FtsZ**  the  **FtsA**  protein and a globular part of the SpoIIE protein were identified.  Ref: 11830939 Folia Microbiol Praha , 2001
- It comprises 15 open reading frames, of which the organization is mraZ mraW ftsL ftsI murE murF mraY murD ftsW murG murC ftsQ  **ftsA**   **ftsZ**  envA, in that order.  Ref: 12153713 J Biochem Tokyo , 2002
- These localization results support the model that E. coli cell division proteins assemble sequentially as a multimeric complex at the division site first  **FtsZ** , then  **FtsA**  and ZipA independently of each other, followed successively by FtsK, FtsQ, FtsL, FtsW, FtsI and FtsN.  Ref: 11703663 Mol Microbiol, 2001
- Since these results remain unexplained, we examined an  **ftsZ**  mutant and other cell division mutants  **ftsA** , ftsQ, and ftsI and a PBP2 mutant for induction of beta lactamase.  Ref: 12107141 J Bacteriol, 2002
- septal localization of FtsW required  **FtsZ** ,  **FtsA** , FtsQ, and FtsL but not FtsI.  Ref: 11807049 J Bacteriol, 2002
- ZipA and  **FtsA**  are recruited independently to the  **FtsZ**  cytokinetic ring Z ring and are essential for cell division of Escherichia coli.  Ref: 12634424 Proc Natl Acad Sci U S A, 2003
- Specifically, cephalexin acylated PBP3 about 50% faster in a population of dividing cells than in a population of filamentous cells in which division was inhibited by inactivation or depletion of  **FtsZ** ,  **FtsA** , FtsQ, FtsW, or FtsN.  Ref: 12813065 J Bacteriol, 2003
- Consistent with a direct interaction between  **FtsZ**  and the MinCD inhibitor, we find that increased  **FtsZ** , but not  **FtsA** , suppresses MinCD induced lethality.  Ref: 11673433 J Bacteriol, 2001
- These regions of actin and tubulin, which are not present in their prokaryotic counterparts  **FtsA**  and  **FtsZ** , are involved in the polymerization of the two cytoskeletal proteins.  Ref: 11580270 J Struct Biol, 2001
- In Escherichia coli, at least nine essential gene products participate directly in septum formation  **FtsA** , FtsI, FtsL, FtsK, FtsN, FtsQ, FtsW,  **FtsZ**  and ZipA.  Ref: 11703663 Mol Microbiol, 2001
- Observation of cell morphology revealed that the coexpression of the  **ftsA**  and  **ftsZ**  genes successfully suppressed filamentation caused by the accumulation of recombinant proteins.  Ref: 12571061 Appl Environ Microbiol, 2003
- Consistent with this, we found that an  **FtsZ**  mutant unable to interact with both ZipA and  **FtsA**  was unable to assemble into Z rings.  Ref: 11847116 EMBO J, 2002
- A region of the dcw division cell wall cluster, including ftsQ,  **ftsA** ,  **ftsZ**  and murC, was sequenced in DX and SIN strains as a basis for studying cell division.  Ref: 12429070 BMC Microbiol, 2002
- The results imply that association of both  **FtsA**  and ZipA with  **FtsZ**  polymers is needed for further maturation of the nascent organelle.  Ref: 11948172 J Bacteriol, 2002
